# Targeting the Endothelin Axis with Atrasentan, in combination with IFN-alpha, in metastatic renal cell carcinoma

**DOI:** 10.1038/bjc.2011.515

**Published:** 2012-01-03

**Authors:** G Groenewegen, M Walraven, J Vermaat, B de Gast, E Witteveen, R Giles, J Haanen, E Voest

**Affiliations:** 1 Department of Medical Oncology, University Medical Center Utrecht, Heidelberglaan 100, Utrecht 3584 CX, The Netherlands; 2 Department of Medical Oncology, VU University Medical Center, De Boelelaan 1117, Amsterdam 1081 HV, The Netherlands; 3 Department of Medical Oncology, The Netherlands Cancer Institute, Plesmanlaan 121, Amsterdam 1066 CX, The Netherlands; 4 Department of Nephrology and Hypertension, University Medical Center Utrecht, Heidelberglaan 100, Utrecht 3584 CX, The Netherlands

**Keywords:** atrasentan, IFN-α, endothelin axis, mRCC, VEGF

## Abstract

**Background:**

The endothelin system is involved in tumour growth. Atrasentan, a selective endothelin-A-receptor antagonist, blocks endothelin signalling. This phase I trial studied combining treatment of interferon-alpha (IFN-*α*) with atrasentan in renal cell carcinoma (RCC).

**Patients and methods:**

This study evaluated the safety and tolerance of IFN-*α* (9MU subcutaneously (s.c.) three times a week) in combination with atrasentan (2.5, 5 and 10 mg orally once daily) in untreated metastatic RCC. Cohort 10 mg was extended to obtain insights in efficacy and pharmacodynamics.

**Results:**

Observed toxicities mainly consisted of known IFN-like toxicities (anorexia, chills, fever, fatigue and nausea), and of nasal congestion (associated to atrasentan). None of these toxicities were considered dose limiting. Cohort 10 mg was extended up to 32 patients; in a subset of patients treated according to the protocol (*n*=27), median overall survival (OS) was 17.3 months. One patient (3.1%) showed a partial response lasting 14.3 months. In an exploratory analysis, we observed that in the subset of patients with declining vascular endothelial growth factor (VEGF) levels (in combination with rising Endothelin-1 levels), median OS was 22.2 months compared with 2.2 months in patients with increasing VEGF levels.

**Conclusion:**

Combination treatment of IFN-*α* 9MU-*α* s.c. three times a week and atrasentan 10 mg once daily is tolerated. Clinical activity, especially OS, and biomarkers in our view warrant further studies targeting the endothelin axis.

Metastatic renal cell carcinoma (mRCC) has been subject to many trials with different approaches, including immunotherapy, chemotherapy and targeted therapy. Until recently and at the moment of designing this study, interferon-alpha (IFN-*α*) was standard care. This cytokine provided survival benefit over supportive therapy (Coppin, 2008) or hormone therapy with medroxyprogesterone acetate (Medical Research Council Renal Cancer Collaborators, 1999). IFN-*α*-induced toxicity largely consists of flu-like symptoms (Coppin, 2008). Although its anti-cancer effects are still not comprehensively understood, anti-proliferative, anti-angiogenic and immunogenic characteristics have been described (Kirkwood, 2002). The present treatment options, consisting of sunitinib, sorafenib and bevacizumab, address the vascular endothelial growth factor (VEGF) pathway (Motzer *et al*, 2007; Escudier *et al*, 2007a, 2007b).

The target of the current study is the endothelin axis. Endothelin-1 (ET-1) is primarily expressed by endothelial cells but also by cells of certain types of cancer (Nelson *et al*, 2003). ET-1 promotes the production of VEGF (Ferrara, 2004), through enhanced HIF-1*α* stability and accumulation (Spinella *et al*, 2002). Furthermore, ET-1 is mitogenic to cancer cells, and has anti-apoptotic and tumour-enhancing effects (Spinella *et al*, 2002; Nelson *et al*, 2003; Ferrara, 2004; Bagnato and Rosano, 2008), mediated by the endothelin-A-receptor (ET_A_R) (Pflug *et al*, 2007). Receptor blockade by specific antagonists inhibits the proliferative effects of ET-1 in cancer cells (Nelson *et al*, 2003) and reverses ET-1-mediated cell survival during chemotherapy-induced apoptosis (Pflug *et al*, 2007).

Atrasentan (ABT-627) was developed as a potent, selective ET_A_R inhibitor (Opgenorth *et al*, 1996). Phase I studies reported maximum tolerated doses of 60 mg (Carducci *et al*, 2002), 75 mg (Ryan *et al*, 2004) or 95 mg (Zonnenberg *et al*, 2003) once daily. Most side effects were attributable to the vasoactive nature, including rhinitis, headache, peripheral oedema and anaemia (caused by hemodilution). On the basis of pharmacodynamic characteristics, the dose of 10 mg once daily was chosen for further studies, mostly performed in prostate cancer (Carducci *et al*, 2003, 2007).

Because ET-1 and ET_A_R are overexpressed in RCC (Douglas *et al*, 2004; Pflug *et al*, 2007), suggesting pathological endothelin signalling, and because of complementary mechanisms of action exercised by atrasentan and IFN-*α*, we hypothesised that mRCC patients might benefit from combining these agents. This phase I study evaluates the safety of combination treatment with atrasentan 10 mg once daily and IFN-*α* 9MU subcutaneously (s.c.) three times a week. An extension cohort was included to obtain an insight into toxicities in a larger set of patients, and to obtain an insight in anti-tumour effects and pharmacodynamics in an exploratory analysis of VEGF and ET-1 kinetics.

## Patients and methods

### Study design

This open-label phase I study protocol was reviewed and approved by Institutional Review Boards of the participating centres and conducted according to institutional, national and European guidelines. Patients were required to provide written informed consent before study participation. This study was not registered in a trial database, as this was not common practise at the time of initiation. Patient enrolment was between January 2003 and May 2007.

#### Dose escalation

The primary objective of the dose escalation part, performed by the University Medical Center Utrecht only, was to demonstrate the safety and tolerability of combination treatment of IFN-*α* (IFN-*α*-2a, Roferon, Roche; Roche Nederland, Woerden, The Netherlands) 9MU s.c. three times a week and atrasentan (Abbott Laboratories, Chicago, IL, USA) 10 mg orally once daily. A fixed dose of IFN-*α* was combined with escalating doses of atrasentan (2.5, 5 or 10 mg once daily) in three predefined consecutive cohorts, according to a standard design of 3–6 patients per cohort. Atrasentan was started 2 weeks after the first administration of IFN-*α*. Dose escalation was based on at least three assessable patients per dose level completing 4 weeks of the combination treatment. Dose limiting toxicity (DLT) was defined as grade 3/4 adverse events occurring in the first 4 weeks of combination treatment, except for nausea, vomiting and fever. Standard supportive care consisted of anti-pyretics (acetaminophen) and anti-emetics (metoclopramide).

#### Extension of cohort 10 mg

The objective of the extension of cohort 10 mg, performed at two centres, was to obtain better insight into the toxicity and to obtain information on progression-free survival (PFS), overall survival (OS) and biomarkers dynamics. All patients received 10 mg atrasentan once daily and the fixed dose of IFN-*α* as in the phase 1 dose escalation part.

Toxicity evaluation was performed according to Common Terminology Criteria for Adverse Events version 3.0 (CTCAE). Toxicities were clustered into treatment periods. Period 1 included the 2 weeks of IFN-*α* monotherapy, period 2 the first 4 weeks of combination treatment and period 3 the episode of continued treatment thereafter.

**Figure 1:**
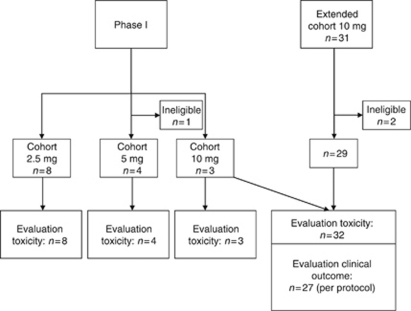
Patient enrolment and conduct of study.

Treatment was continued for 1 year or until unacceptable toxicity or progressive disease (PD) was reported. PD was assessed according to Response Evaluation Criteria in Solid Tumours (RECIST). PFS and OS were recorded from start of IFN-*α* treatment until documented PD or death. Clinical outcome was assessed in the patients treated per protocol (exposed to combination treatment), as depicted in Figure 1. Toxicity was evaluated in the intent-to-treat group (receiving at least one dose of IFN-*α*).

### Patients

Patients with histologically confirmed mRCC (including non-clear cell) without prior systemic treatment were included. Tumour progression had to be demonstrated within 6 weeks before the start of study treatment. Other key eligibility criteria included age >18 years, life expectancy of more than 3 months, WHO performance status score of 0–2 and measurable disease. Adequate renal, hepatic, bone marrow and cardiac functions were required, and any hypertension needed to be controlled. Exclusion criteria were primary tumours other than RCC, clinical evidence of cerebral metastases, immune disease, active infection, use of immunosuppressive and/or non-steroidal anti-inflammatory drugs, HIV positivity, pregnancy or lactation. Radiotherapy had to be completed at least 4 weeks before the administration of study medication.

### Assessments

Patient visits were planned pre-treatment, once a week during the first 6 treatment weeks, bi-weekly for the next 6 weeks, then every 4 weeks until 36 weeks, followed by every 2 months until treatment termination. Study procedures consisted of physical examination and laboratory assessments. Tumour measurements were performed every 8 weeks on-treatment, with appropriate imaging techniques. After treatment discontinuation, survival evaluation and PD assessment occurred every 3 months for 1 year, thereafter every 6 months.

### Pharmacodynamic analyses

For an exploratory analysis, plasma samples and platelet counts were obtained pre-treatment, and 2, 4 and 8 weeks after the first administration of IFN-*α*. Plasma ET-1 and VEGF concentrations were quantified in 27 of 44 patients, using commercially available ELISA kits (R&D Systems, Minneapolis, MN, USA) following manufacturer's protocols.

### Statistical analysis

Time-to-event analyses were performed using the Kaplan–Meier methodology.

Paired *t*-tests were performed to evaluate the changes in biomarker levels over time. The log rank test was used to correlate biomarkers with PFS/OS. Platelet counts were correlated to VEGF levels with the Pearson correlation coefficient.

For these analyses SPSS (version 15.0; SPSS INC., Chigaco, IL, USA) was used with defined significance level of P<0.05. Reported *P*-values are two-sided.

## Results

### Conduct of the study

A total of 16 patients were included in three dose escalation cohorts of the phase I study (Figure 1). The total number of patients of cohort 2.5 mg was eight. Because of a DLT (pulmonary embolism) in one patient this cohort was extended. One patient did not complete 4 weeks of combination treatment because of hepatic toxicity ascribed to IFN-*α*; this patient was replaced. Unintentionally, the first patient of cohort 5 mg received 2.5 mg atrasentan instead of 5 mg once daily; this patient was included in cohort 2.5 mg.

One patient of cohort 5 mg was ineligible for analysis, as the pathologist's review diagnosed metastatic colorectal cancer. Another patient of this cohort discontinued treatment because of cerebral metastases, which became clinically manifested at an early stage of treatment; this patient was replaced and still included in the safety analysis. DLT's were not observed in this cohort.

The three initial patients in cohort 10 mg did not show any DLT. A total of 31 patients were enroled in the extension of cohort 10 mg (Figure 1). Two patients were deemed ineligible because of inadequate performance status and of unconfirmed histological diagnosis; these patients were excluded from the analysis. Combining these numbers, the total number of patients evaluated for toxicity was 32.

Of these 32 patients, 5 did not start atrasentan treatment because of IFN-*α* toxicity (including 1 allergic reaction), leaving 27 patients in the per protocol group. Patient enrolment in the study was discontinued once sunitinib became available in the Netherlands for first line treatment of mRCC.

### Patient characteristics

Gender, age and MSKCC risk scores of the patient groups, as depicted in Table 1, were consistent with recent published studies for RCC (Motzer *et al*, 2007; Escudier *et al*, 2007b).

### Treatment duration

Results per patient concerning treatment duration and discontinuation, PFS and OS are presented in Table 2.

#### Phase I

Median time on study treatment for the eligible patients in the escalation part of the study was 163 days (range: 23–364), evaluated from the start of IFN-*α* administration. Two patients in cohort 2.5 mg discontinued treatment because of toxicity, three because of PD and three because of completion of 1 year of treatment. The discontinuation in cohort 5 mg was caused by toxicity in one patient and PD in two patients. One patient completed 1 year of treatment. One patient in this cohort required a 33% dose reduction of IFN-*α*, following treatment interruption for 1 week, 2 months after start. In cohort 10 mg two patients discontinued as a consequence of PD, while one patient completed 1 year of treatment.

#### Extended cohort 10 mg

Median time on study treatment in the entire group (*n*=32) and the treated per protocol group (*n*=27) was 68 days (range: 1–364) and 102 days (range: 20–364), respectively. One patient withdrew the informed consent after 1 month of treatment and one patient discontinued treatment because of irradiation of remaining lesions, which had regressed on study treatment. For this patient PFS was censored from the start of radiation treatment. One patient discontinued treatment because of PD, however, retrospectively this patient had PD earlier during treatment. Other treatment discontinuations were due to completion of 1 year of treatment (*n*=2), PD (*n*=16) and toxicity (*n*=12). From the latter 12 patients, 5 received <2 weeks of treatment (only IFN-*α*), 3 patients 3–4 weeks, 3 patients approximately 2.5 months (1 patient received only 2 weeks atrasentan, but continued with IFN-*α*) and 1 patient 6 months.

Three patients discontinued IFN-*α* prematurely after 2, 4 and 5.5 months, but continued atrasentan for an additional 4, 2 and 1 month, respectively. Five patients halted atrasentan treatment after 3 weeks, 1.5 months, 1.5 months, 2 months and 8 months of study start, but continued IFN-*α* for an additional 1.5 months, 1 week, 3 weeks, 3 weeks and 2 months, respectively.

### Toxicity: phase I plus extension of cohort 10 mg

Table 3 shows grade 1/2 treatment-related toxicity when observed in more than one patient and all grade 3/4 adverse events of both phase I study, and the extension of cohort 10 mg. The three atrasentan dose levels were combined, as the occurrence of adverse events was similar both quantitatively and qualitatively (data not shown). All reported non-laboratory adverse events were grade 2 or less, except for fever (excluded as DLT), allergic reaction and pulmonary embolism. Notably, the most adverse events began in period 1 (IFN-*α* monotherapy). Flu-like symptoms generally diminished over time. Three patients were hospitalised with fever, most likely attributed to IFN-*α*, accompanied by anorexia/vomiting in two patients. One patient was hospitalised because of an allergic reaction to IFN-*α*. Three patients had complaints of dyspnoea, weight loss, malaise, nasal congestion or fatigue, resulting in the discontinuation of atrasentan while IFN-*α* treatment was sustained. Although vasovagal complaints/dizziness were reported by several patients, no relation to hypotension could be established as changes in blood pressure were not observed (data not shown). One hypertensive patient discontinued anti-hypertensive treatment after the start of study treatment and remained normotensive throughout the study. Headaches were handled adequately with acetaminophen.

The observed laboratory abnormalities were as expected from IFN-*α* or atrasentan treatment. Approximately 85% of patients developed grade 1/2 anaemia during period 2 (first 4 weeks of combination treatment), recovering thereafter. Neutropenia and lymphocytopenia, observed during long-term treatment, did not result in infection.

### Clinical outcome measures: per protocol group treated with 10 mg once daily atrasentan

Data cut-off was set at the first of August 2010. In Table 4, median OS and PFS are presented, including data for the separate risk groups (the MSKCC index was also used for patients with non-clear histology RCC) (Motzer *et al*, 2002). Median and PFS in the per protocol group were 17.3 and 5.1 months, respectively. Best OS of 30 months was observed in the best risk subset.

Eight patients treated with 10 mg atrasentan received treatment with sunitinib or sorafenib upon disease progression. If censored for this treatment, median OS extended to 19.8 months. One patient (3.1%) showed a partial response lasting 14.3 months.

### Biomarkers

An exploratory analysis was performed for biomarkers. VEGF expression is an established biomarker associated with worse prognosis in RCC and other tumour types (Djordjevic *et al*, 2007; Dirim *et al*, 2008). We reasoned that VEGF and ET-1 levels might serve as potential biomarkers in this study. In an exploratory analysis, 27 of 44 patients were categorised into three groups, according to baseline VEGF levels and dynamics during treatment (Table 4). Group 1 (*n*=16) showed low plasma baseline VEGF levels, which remained low throughout treatment. Group 2 (*n*=7) showed elevated baseline levels, which significantly declined during treatment. Finally, in group 3 (*n*=4), initially elevated levels further increased during treatment.

**Figure 2:**
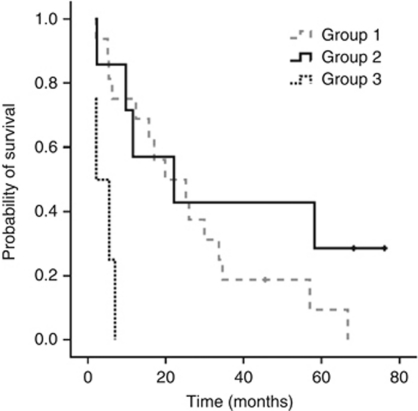
Kaplan–Meier estimates of OS of groups categorised to VEGF level dynamics (definition of groups is outlined in the text and in Table 4).

ET-1 levels significantly increased in group 1 and group 2 (Table 5). However, ET-levels did not change in group 3. Survival analysis was explored for these three groups (Figure 2 and Table 4). Best OS and PFS were observed in groups 1 and 2.

As platelets are the main transporters of VEGF in the blood (Verheul *et al*, 1997) and thrombocytosis is related to tumour progression (Symbas *et al*, 2000; Bensalah *et al*, 2006), platelet counts were analysed during treatment. Platelet counts appeared to be related to plasma VEGF concentrations (Pearson's correlation coefficient, *r*=0.64, *P*=0.01). It is therefore tempting to speculate that platelets might influence tumour biology. Further exploration of this observation is ongoing.

## Discussion

This is to our knowledge the first reported study to target the endothelin axis in RCC. In our view, it shows that combination treatment with 9MU IFN-*α* s.c. three times a week and 10 mg atrasentan orally once daily in previously untreated mRCC patients is well tolerated and seems to show biological and clinical activity.

Observed toxicities (anorexia, chills, fever, fatigue, nausea and nasal congestion) were manageable with supportive care and in accordance with previous studies on IFN-*α* or atrasentan. None of the toxicities were considered dose limiting. The majority of adverse events originated in the first 2 weeks of IFN-*α* treatment, before the administration of atrasentan. The number of toxicity-related discontinuations (34% of patients) could be largely attributed to IFN-*α*, and is comparable to a recent trial combining bevacizumab with IFN-*α* (Escudier *et al*, 2007b). Toxicity profiles of IFN-*α* and atrasentan did not seem to influence each other.

The cohort 10 mg was extended in order to study toxicity more extensively and to obtain a first impression of the clinical activity of this combination. Observed efficacy results showed a median OS of 17.3 months in the per protocol group and 30 months in the best risk group, which is higher than Motzer's defined clinical outcome expectations for IFN-*α* monotherapy in mRCC (Motzer *et al*, 2002). We are fully aware that inter-study comparison of clinical outcome is full of limitations. These results suggest an effect of atrasentan on top of IFN-*α*. Further clinical studies are needed to clarify this effect. Collectively, combination treatment with 9MU IFN-*α* s.c. three times a week and 10 mg atrasentan orally once daily is tolerated and our data tentatively indicate the notion that this combination might induce a more indolent course of disease, possibly contributed by additive and/or synergistic anti-tumour effects of both the agents.

VEGF is an important target in RCC treatment. IFN-*α* treatment recently showed to inhibit VEGF expression or secretion in several tumour types *in vitro* and *in vivo* (Rosewicz *et al*, 2004; Wu *et al*, 2005; Raig *et al*, 2008; Tochizawa *et al*, 2008). Moreover, ET_A_R blockade reduced the promotion of VEGF production (Rosano *et al*, 2003; Gorenflo *et al*, 2007). We observed that, although the pre-treatment VEGF levels of patients in group 2 were elevated, the subsequent drop resulted in a clinical outcome that was similar to group 1 with low VEGF concentrations and the best OS. Although exploratory in nature and based on only a small number of patients, these results are of interest because, to our knowledge, no association between reduction of increased pre-treatment VEGF levels and improved clinical outcome has been previously described. We also observed increased ET-1 levels in group 1 and 2 during treatment. If receptor blocking results in increased levels of the ligand, as observed in other studies as well (Carducci *et al*, 2002; Zonnenberg *et al*, 2003), this supports a role of atrasentan in these observed pharmacodynamic effects. Further studies will need to clarify these putative effects of atrasentan.

As to how to proceed, the results of this study in our view warrant further exploration of the endothelin axis as a target in mRCC treatment. For a phase II study, a randomised discontinuation trial design for atrasentan with continuous IFN-*α* or a randomised study with IFN-*α* with and without atrasentan would be the appropriate study designs. However, as the standard of care has changed from IFN-*α* to sunitinib, these options are not realistic. Also, a randomised study comparing combination treatment IFN-*α* and atrasentan with the current standard of care sunitinib is unlikely to be sufficiently supported in the field. Alternatively, atrasentan could be combined with other targeted drugs, particularly VEGF and mTOR inhibitors. This seems especially feasible, as the mild toxicity profiles for atrasentan as a single agent and in combination with interferon suggest a tolerable combination with other targeted drugs. Particularly, the VEGF-reducing activity and vasoactive nature make it an attractive candidate for combination treatment with TKIs.

In conclusion, we present a study combining atrasentan with IFN-*α* in patients with previously untreated progressive mRCC showing tolerability and clinical anti-tumour effects. Clinical activity seems to be related to low VEGF levels (either low levels throughout treatment or elevated baseline levels that declined during treatment) and to increasing ET-1 levels. These findings warrant further exploration of targeting the endothelin axis in mRCC.

## Figures and Tables

**Table 1:** Baseline demographics and clinical characteristics

**Characteristics**	**Phase 1 (*n*=15)**	**Extended cohort 10 mg (*n*=32)**	**All patients (*n*=44)**
*Sex, no. (%)*			
Male	13 (87)	22 (69)	32 (73)
Female	2 (13)	10 (31)	12 (27)

Median age year (range)	61 (37–73)	60 (35–76)	59 (37–76)
			
*WHO ECOG performance status, no. (%)*			
Zero	9 (60)	18 (56)	24 (54.5)
One	4 (27)	11 (34)	15 (34)
Two	1 (6.5)	1 (3)	2 (4.5)
Unknown	1 (6.5)	2 (6)	3 (7)
			
*Tumour histologic type, no. (%)*			
Clear cell	9 (60)	27 (84)	34 (77)
Other/not specified	6 (40)	5 (16)	10 (23)

Previous nephrectomy, no. (%)	10 (67)	23 (72)	31 (70)
Progressive disease, no. (%)	15 (100)	32 (100)	44 (100)

*MSKCC risk score, no. (%)*			
Favarouble	6 (40)	11 (34)	16 (36)
Intermediate	8 (53)	18 (56)	25 (57)
Poor	1 (7)	3 (9)	3 (7)

Abbreviations: ECOG=Eastern Cooperative Oncology Group; MSKCC=Memorial Sloan-Kettering Cancer Center; WHO=World Health Organization.

Percentages may not be a total of 100 because of rounding.

**Table 2:** Results per patient concerning treatment duration and discontinuation, PFS and OS

**Cohort**	**Patient**	**Treatment duration (mo)**	**PFS (mo)**	**OS (mo)**	**Reason of discontinuation**	**Alive**	**Sunitinib/sorafenib**
2.5 mg	1	11	15	21.1	1 year treatment		
	2	7.7	7.7	8.3	PD		
	3	0.6	0.6	1.8	Toxicity		
	4	0.8	1.3	5.5	DLT		
	5	12	27.2	57.1	1 year treatment		
	6	3.5	3.5	5.4	PD		
	7	1.8	1.8	20.8	PD		
	8	12	21.6	58.3	1 year treatment		Yes
5.0 mg	9	12.1	24	66.7	1 year treatment		
	10	1	1	2.2	PD		
	11	11.3	12.3	76.1	Toxicity	Yes	Yes
	12	2	2	9.6	PD		
10.0 mg	13	12.1	22	68.3	1 year treatment	Yes	Yes
	14	3.4	3.4	22.1	PD		
	15	5.4	5.4	6.3	PD		
10+	16	5.8	5.8	25.2	PD		
	17	11.5	7.5	26	PD		
	18	2.1	2.1	5.2	PD		
	19	2.2	5.2	30	Toxicity		
	20	0.4	0.4	2.3	Toxicity		
	21	0.7	2.8	13.7	Toxicity		
	22	2.2	2.2	17.1	PD		
	23	11.2	18.3	34.6	1 year treatment		
	24	5.8	5.8	12.4	PD		
	25	6	6	33.7	PD		Yes
	26	6.1	6.1	11.6	PD		
	27	0.9	1.3	2	Toxicity		
	28	9.9	9.9	16	PD		Yes
	29	0.1	0.1	0.7	Toxicity		
	30	2.2	2.2	5.4	PD		
	31	0.7	0.7	2.4	Toxicity		
	32	0.5	1.4	7	Toxicity		Yes
	33	5.9	5.9	45.5	Toxicity	Yes	Yes
	34	0.2	0.7	2.2	Toxicity		
	35	2	2	15.7	Toxicity		Yes
	36	2.5	6.9	19.9	Toxicity		Yes
	37	2.3	2.3	17.3	PD		
	38	3.7	3.7	19.8	PD		
	39	5.1	5.1	30.2	PD		
	40	1.8	1.8	12	PD		
	41	6.2	16.4	48.8	Radiotherapy	Yes	
	42	0	9.9	26.3	Toxicity		Yes
	43	1.4	1.4	3	PD		
	44	0.9	5.2	24.1	Withdrew consent		

Abbreviations: DLT=dose-limiting toxicity; OS=overall survival; PD=progressive disease; PFS=progression-free survival.

**Table 3:** Overview of laboratory and non-laboratory adverse events

	**Period 1 (*n*=44)**		**Period 2 (*n*=39)**		**Period 3 (*n*=33)**	
**Patient number CTC grade^a^**	**1/2**	**3/4**	**1/2**	**3/4**	**1/2**	**3/4**
Abdominal discomfort	2/1		3/1		5/3	
Allergic reaction		1/−				
Alopecia	1/−				3/−	
Anorexia	14/11		9/2		8/1	
Arrhythmia	2/−					
Bleeding	−/2		−/2		2/−	
Chills	27/11		7/3		6/1	
Constipation	−/2				2/1	
Cough	3/3		6/3		5/1	
Dehydration	5/−		6/−		3/1	
Diarrhoea	6/−		3/−		5/−	
Dizziness	5/1		1/1		2/2	
Dyspnoea			5/4		5/2	
Oedema limb			10/−		3/−	
Fatigue	20/11		9/10		6/5	
Fever	12/8	1/−	1/2		2/−	1/−
Headache	12/−		2/−		1/−	
Insomnia	2/1		1/2		2/1	
Malaise	6/6		1/5		3/−	
Mood alteration	4/−		2/−		−/4	
Nasal congestion	4/−		17/2		8/−	
Nausea	14/2		10/1		5/2	
Neuropathy	−/1				−/2	
Pulmonary embolus						−/1
Skin problems	2/−		9/2		10/2	
Tumour pain	3/2		3/2		8/3	
Vasovagal period			2/1		1/−	
Voixe changes					7/1	
Vomiting	2/−		4/−			
Weight loss	8/−		3/2		2/1	
Increased total bilirubin		−/3				
Increased alkaline phosphatase	3/−	2/−	6/2	1/−	7/−	
Increased gamma-glutamyltranferase	15/7	1/1	16/7	3/1	10/5	2/−
Increased aspartate aminotransferase	13/1		14/3		10/−	
Increased alanine aminotransferase	9/1		14/2		4/2	
Increased creatinine	18/1		16/1		14/2	
Anaemia	18/7		21/11	1/−	12/12	2/−
Trombocytopenia	5/−		8/−		8/−	
Leucopenia	8/3		12/9		9/8	2/−
Neutropenia	−/2		2/4	1/−	4/4	1/−
Lymphocytopenia	1/12	3/−	5/12	6/−	2/13	7/−
Hyponatriema	11/1	1/−	11/1	1/−	8/−	1/−
Potassium disregulation	11/1		12/1	1/−	6/1	
Calcium disregulation	13/−		19/−		17/2	−/2
Hypoalbuminemia	7/4		14/6		11/3	

Listed are all laboratory and non-laboratory adverse events (for grade 1/2 only when observed in more than one patient). All severity was graded according to the National Cancer Institute.

Period 1: the 2 weeks of IFN-á monotherapy, period 2: the first 4 weeks of combination treatment, period 3: the episode thereafter.

a Common Terminology Criteria for Adverse Events, version 3.0.

**Table 4:** Summary of the efficacy measures of median overall survival and median progression-free survival of the per protocol group (treated with10 mg atrasentan) and of groups categorised according to VEGF level dynamics

	**Median PFS mo (95% CI)^a^**	**Median OS mo (95% CI)^b^**
*Extended cohort 3, per protocol (n=27)*		
Favourable (*n=*10)	5.1 (2.3–7.9)	17.3 (10.9–23.7)
Intermediate (*n=*15)	5.2 (4.8–5.5)	30.0 (10.0–49.9)
Poor (*n=*2)	3.5 (0.5–6.5)	16.0 (8.4–23.6)
	2.0	5.4
		
*Groups categorised to VEGF levels*		
Group 1 (stable) (*n=*16)	5.3 (4.5–6.1)	19.9 (3.9–35.8)
Group 2 (decreasing) (*n=*7)	6.1 (0–13.5)	22.1 (0–49.1)
Group 3 (increasing) (*n=*4)	1.3 (1.0–1.7)	2.2 (0–5.7)

Abbreviations: CI=confidence interval; OS=overall survival; PFS=progression-free survival; VEGF=vascular endothelial growth factor.

a *P*-values PFS: per protocol=0.317 (favourable+intermediate *vs* poor), groups categorised to VEGF level dynamics=0.002 (group 1+2 *vs* 3).

b *P*-values OS: per protocol=0.016 (favourable+intermediate *vs* poor), groups categorised to VEGF level dynamics=0.000 (group 1+2 *vs* 3).

**Table 5:** Plasma levels (pg ml^−1^) of VEGF and ET-1 before (baseline) and during study treatment (t=4 weeks) of groups categorised to VEGF level dynamics, presented as median and range of each group

	**Group 1 (*n*=16)**	**Group 2 (*n*=7)**	**Group 3 (*n*=4)**
*Median VEGF levels (range) (pg ml* ^*−1*^ *)*			
Baseline	36.4 (5.6–71.8)	248.9 (103.7–476.9)	119.9 (41.9–628.5)
t=4 wk	21.8 (7.2–61.7)	45.5 (32.7–152.0)	262.9 (162.9–691.6)
*P*-value^*^	>0.15	0.016	0.045
			
*Median ET-1 levels (range) (pg/ml)*			
Baseline	3.2 (0.3–5.0)	3.3 (1.9–4.9)	2.9 (1.8–4.9)
T=4wk	4.9 (2.9–7.5)	6.1 (3.8–7.9)	2.6 (0.3–6.7)
*P*-value^*^	0.001	0.006	>0.15

Each sample was analysed in duplicate; with a coefficient of variation for the assay of 9.4%. Mean of duplicate was used for further calculations.

^*^P-values: paired *T*-test baseline vs T=4 weeks.
